# Reliability of the entomovector technology using Prestop-Mix and *Bombus terrestris* L. as a fungal disease biocontrol method in open field

**DOI:** 10.1038/srep31650

**Published:** 2016-08-17

**Authors:** Reet Karise, Gerit Dreyersdorff, Mona Jahani, Eve Veromann, Eve Runno-Paurson, Tanel Kaart, Guy Smagghe, Marika Mänd

**Affiliations:** 1 Institute of Agricultural and Environmental Sciences, Estonian University of Life Sciences, Kreutzwaldi 1, 51014 Tartu, Estonia; 2 Department of Crop Protection, Ghent University, Coupure links 653, B-9000, Ghent, Belgium; 3 Institute of Veterinary Medicine and Animal Sciences, Estonian University of Life Sciences, Kreutzwaldi 62, 51014 Tartu, Estonia

## Abstract

*Botrytis cinerea* Pers.:Fr. is a major plant pathogen, and a new approach is needed for its control in strawberry to minimise the increasing use of synthetic fungicides. The biofungicide Prestop-Mix, which contains *Gliocladium catenulatum*, is effective against *Botrytis* infections; however, the need for frequent applications increases the costs for farmers. Here, we demonstrate that bumble bees, *Bombus terrestris* L., effectively disseminate the preparation onto flowers in open field conditions. Over the course of three years, we found a highly significant decrease in the rate of *Botrytis* infection. Pathogen control was achieved with relatively low numbers of *G. catenulatum* spores per flower, even using flowers that are not highly attractive to bumble bees. An even distribution of spores was detected up to 100 m from the hives, either due to primary inoculation by bumble bees or secondary distribution by other flower visitors such as honey bees and solitary bees. We showed that the application of a biocontrol agent by bumble bees is reliable for the use of environmentally friendly pest control strategies in northern climatic conditions. This low cost technology is especially relevant for organic farming. This study provides valuable information for introducing this method into practice in open strawberry fields.

Strawberry, *Fragaria x ananassa* Duch., is one of the most important berry crops worldwide. The production of strawberries is heavily affected by fungal diseases, among which the grey mould caused by *Botrytis cinerea* Pers.:Fr. is suspected to cause high yield losses^1^. The protection against grey mould has been achieved by synthetic fungicide use; however, there is a risk of plant pathogens developing high levels of fungicide resistance^2^,^3^, and pesticide residues may remain in the fruits^4^. Moreover, organic farming practices need alternative grey mould control methods to retain their competitiveness in the market.

Prestop-Mix (Verdera OY, Finland) is a biofungicide that contains mycelia and spores of a soil fungus, *Gliocladium catenulatum* Gilman & E. Abbot Strain J1446. This fungus efficiently controls plant diseases caused by *Pythium, Rhizoctonia, Fusarium* and *Botrytis* fungi^5^. The application of Prestop-Mix through spraying is laborious and might not provide full control^6^. Spraying this product dissolved in a water solution only achieves the desired effect when plants are repeatedly treated because the biocontrol agent has to reach the flowers, where most of the infection occurs^7^. In addition, the spraying technique is wasteful because it involves covering the entire surfaces of the plants and the surrounding areas such as soil, mulching material or strawberry plant leaves.

The use of bee-vectors that visit flowers as biocontrol agents has been tested for more than 20 years^8^, and this strategy is currently used by farmers^9^. Honey bees, bumble bees and even mason bees have been used for this purpose^8^,^9^,^10^,^11^,^12^,^13^,^14^. The use of foraging bees as disseminators of biopesticides guarantees that the biocontrol agent will reach flowers^15^,^16^,^17^,^18^ as they open. Moreover, the disseminating bees provide an additional pollination service that leads to an increased fruit weight and yield^19^,^20^,^21^.

The bee-vectoring system involves several interactions between the vector, the target crop and the pest organism^22^. Compared to a greenhouse, open field conditions create several additional obstacles when using bees for preparation delivery. The most important obstacles are the attraction of the disseminating bees to non-target plants, the flower visiting behaviour and the weather conditions. Bumble bees are generalist pollinators that visit several (2–4) plant species within the same foraging trip. According to Somme *et al*.^23^, wild *Bombus terrestris* L. have a lower abundance of foragers (33%) that carry pure pollen loads compared with other bumble bee species. This behaviour means that the preparation may be dispersed to non-target plants rather than to the target crops. Although *B. terrestris* is the main species commercially available in Europe, this issue must be considered when purchasing colonies for the purpose of pollination or entomovectoring services. The chemical composition and amount of pollen and nectar of the target crop significantly affect the flower visiting behaviour of bees^23^,^24^. Weather conditions also affect the foraging activity of all bees. Although bumble bees can forage in relatively poor weather^25^, they may stay in the hive when food resources are plentiful inside. The weather also affects the disease pressure because grey mould can rapidly develop and spread with the help of wind^26^, raindrops^27^ and even insects visiting the plants^28^. When the disease pressure is high, curative applications might not be effective, and only preventive methods could help save the harvest^9^,^15^,^29^.

The attractiveness of strawberry flowers and the dependence on insect pollination varies among cultivars. Similarly, the surrounding plant communities, which affect the bumble bee foraging behaviour, can vary from year to year. Regional differences in environmental conditions within Europe are considerable and region-specific data are needed for agricultural practices. Therefore, a three-year study was conducted in open fields with the ‘Sonata’ strawberry cultivar to estimate (1) the efficacy of bumble bees as vectors of the biofungicide Prestop-Mix for controlling *Botrytis* infection; (2) the rate of attractiveness of strawberry flowers to bumble bees; (3) the dispersal distance over the field and (4) the impact of the extra pollination service on the fruit size.

## Results

### Weather

The weather conditions were notably different among the three studied years. In reference to the long-term average temperature (Table 1), the mean air temperature in May was 1 °C higher in 2012 and 2014, whereas it was 4 °C higher in 2013. In June, the mean average monthly air temperature was almost 1.5 °C cooler in 2012 and 2014, but almost 3 °C higher in 2013. Each year, the temperature in July was higher than the long-term average.

The mean average rainfall in May was higher each year; however, compared with the long-term monthly average, the total precipitation was 145% in 2012, 138% in 2013 and, overall, 170% in 2014. In June, the total monthly precipitation was 128% in 2012, only 51% in 2013 and 193% in 2014. In July, the total monthly precipitation was closer to the long-term average, and it was 92% in 2012, 78% in 2013 and 103% in 2014. The number of days with rainfall (over 1 mm 24 h^−1^) was almost two times higher in 2014 than in 2012 or 2013.

### Biocontrol and pollination services provided by bumble bees

Based on a non-parametric analysis, the quantification cycle (Cq) values per flower (31.8 ± 0.15) over all the distances from the bumble bee hive (0, 25, 50, 75 and 100 m) and all the time periods (4, 5, 6 and 7 days) were significantly equal (distance: *F* = 0.65 *df* = 4 *P* = 0.63; day: *F* = 0.37 *df* = 3 *P* = 0.78; distance x day: *F* = 0.83 *df* = 12 *P* = 0.62). Thus, on average, 12.3 spores of Prestop-Mix were evenly dispersed per flower over the different distances in the field from the distribution point, i.e., the dispenser connected to the bumble bee hive. Similarly, the spores were evenly distributed during the course of the experiment.

We counted 0.13 ± 0.02 bumble bees per 10 m of a transect section, and the number did not significantly change throughout the flowering period (*F*_2,251_ = 2.15 *P* = 0.14). The number of foraging bumble bees was similar at each distance (5, 25, 50, 75, 100 m) from the hive (*F*_4,251_ = 0.81, *P* = 0.53). In addition to bumble bees, there were several other insects found on the strawberry flowers: several fly genera (0.68 ± 0.08 individuals per plot), honey bees (0.46 ± 0.04), solitary bees (0.21 ± 0.03) and syrphid flies (0.10 ± 0.01). The number of these insects did not depend on the distance (all *P* > 0.05).

The pollen gathered by bumble bees contained on average 22.4 ± 1.3% strawberry pollen. A Kruskal-Wallis test showed significant differences among the observations based on the three different years and the two locations (*χ*^2^ = 20.5, *df* = 4, *P* < 0.001). There was one observation that differed from the others: in Polli in 2014, the strawberry pollen forage was extremely low (5.9 ± 2.4%). During the other years and in both locations, the average strawberry pollen forage reached 22.1–25.7% of the total pollen forage, and no significant differences were found among these observations (*χ*^2^ = 2.03, *df* = 3, *P* = 0.56). The strawberry pollen was most extensively gathered during the peak of flowering (29.1 ± 2.1%), whereas, at the beginning of flowering, it was 16.7 ± 2.6% and, at the end, it was 14.9 ± 1.9%. Among all the bumble bee foragers, 18.6% visited only strawberry flowers during one foraging trip.

### Effects of the biocontrol agent on the grey mould infection rate

**Figure 1:**
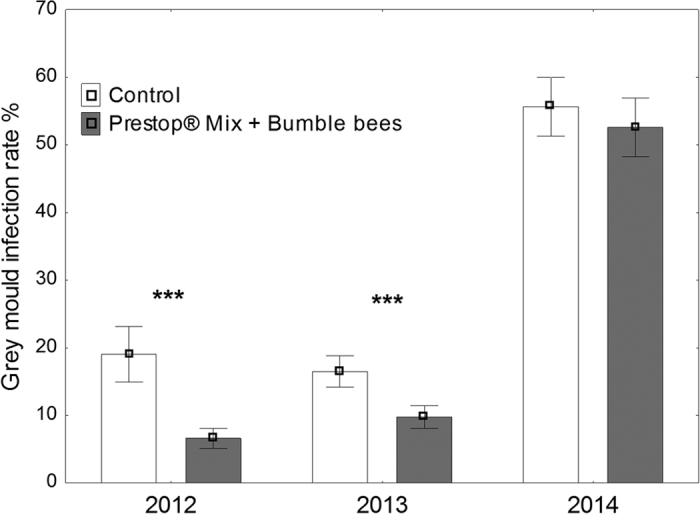
Grey mould infection rate on control plots (not treated) and plots treated with bumble bee-mediated Prestop-Mix. The means with standard error bars are presented. Asterisks (***) indicate a significant decrease (*P* < 0.001) in the infection rate during the years with low pathogen pressure.

The grey mould infection rate in the control plots was different among the observation years (*F*_1,162_ = 1,386.6, *P* < 0.001; Fig. 1). The bumble bee-mediated Prestop-Mix application significantly decreased the grey mould infection (*F*_1,162_ = 132.06, *P* < 0.001). In addition, there was a significant co-effect of the treatment year (*F*_1,162_ = 39.44, *P* < 0.001). A pairwise analysis detected a significant decrease in the grey mould infection rate in the first two years (2012: *t* = −9.30, *df* = 162, *P* < 0.001; 2013: *t* = −8.20, *df* = 162, *P* < 0.001) but not in the third year (2014: *t* = −0.56, *df* = 162, *P* = 0.57).

### Effects of pollination on fruit weight

The mean weight of ten fruits varied widely, ranging from 53.2 ± 3.2 g in Polli (2013) and 192.8 ± 9.8 g in Rõhu (2014). There was no difference in the weight of ten fruits between wind pollinated and open pollinated (wind + bumble bees and other insects) strawberries (*F*_1,250_ = 0.05, *P* = 0.8). The average difference between open pollinated and wind pollinated in the weight of ten fruit samples was 0.8 ± 3.8 g.

### Co-effect of pollination and the biocontrol agent on the total yield

**Figure 2:**
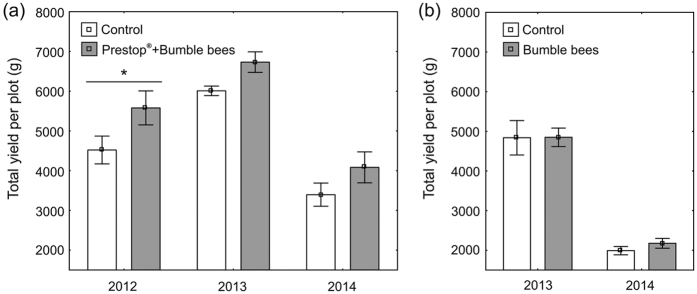
Mean marketable yields of strawberries summarised over the fruit picking period. (**A**) The bumble bee-mediated Prestop-Mix application increased the yield each year. (**B**) Pollination services alone did not change the yield in the cultivar ‘Sonata’. The means with standard error bars are presented. An asterisk (*) indicates significant difference (*P* < 0.05) in the yield between treated and not treated plots.

The bumble bee-mediated Prestop-Mix application significantly affected the total yield (*F*_1,18_ = 9.7, *P* = 0.006; Fig. 2a), whereas the pollination services alone did not (*F*_1,12_ = 0.14, *P* = 0.7; Fig. 2b). The total yield differed among the years (pollination with Prestop-Mix: *F*_1,18_ = 33.31, *P* < 0.001; pollination: *F*_1,12_ = 113.3 *P* < 0.001), although no co-effect of the year and the treatment (*F*_1,18_ = 0.20, *P* = 0.8) nor of the year and the pollination type (*F*_1,12_ = 0.11, *P* = 0.7) was found. The plots with insect pollination and Prestop-Mix treatment produced 123% (Polli, 2012: *t* = 2.32, *df* = 18, *P* = 0.03), 112% (Rõhu, 2013: *t* = 1.58, *df* = 18, *P* = 0.13) and 120% (Rõhu, 2014: *t* = 1.51, *df* = 18, *P* = 0.15) of the yield from control plots (wind pollination and no treatment), whereas the plots with insect pollination alone yielded 100% (Polli, 2013: *t* = 0.03, *df* = 12, *P* = 0.9) and 109% (Polli, 2014: *t* = 0.5, *df* = 12, *P* = 0.6) of the yield from wind-pollinated plots.

## Discussion

Our data confirmed that the bumble bee-mediated biofungicide application is effective for controlling grey mould infections in open field strawberries. However, the efficacy of the treatment depended on the weather conditions. While the infection rate decreased 2.9 and 1.7 times in 2012 and 2013, respectively, no change was observed in 2014 when there was a high pathogen pressure due to heavy rains and cool temperatures. Higher rainfall and colder temperatures most likely favour the growth of grey mould^30^,^31^, and this outcome was confirmed during our experiments. When disease pressure is high, effective control of grey mould is difficult even with chemical fungicides without proper decision-supporting systems^32^. In our experiment in 2014, the monthly rainfall exceeded the long-term average by almost two times in both May and June, whereas the same period in 2012 was cool but relatively dry and in 2013 it was warm and dry. In contrast to our result under high pathogen pressure conditions, effective grey mould control with the same bio-preparation and application method was achieved in a greenhouse experiment by Mommaerts *et al*.^18^. The efficacy of bee-vectored biocontrol was found to be comparable to that of synthetic fungicide spraying^10^.

The bumble bee dispersal over the field was equal, as was the dissemination of BCA on the flowers. According to Mommaerts *et al*.^17^, bumble bees deposit approximately 100 colony forming units (CFUs) per flower in a greenhouse located up to 21 m from the hives with no significant decrease in the CFU numbers. Each bee deposited approximately 23 CFUs to the first flower and approximately 15 CFUs to the second and third flowers. These low numbers can increase after repeated flower visits or decrease after consecutive, unloaded visits of the pollinators. Mommaerts *et al*.^18^ showed that the number of *G. catenulatum* spores per flower needed to effectively control the grey mould might be low because decreasing the amount of MCA to the half of the original did not decrease the control effect observed. The low number of spores counted per flower in our experiment might be the result of differences in the foraging behaviour of pollinators, the crop plant species and the foraging conditions. Bumble bees transported spores of *G. roseum* to raspberry flowers in covered plot conditions with an average of 451–2,461 CFUs/flower^33^. Several aspects might have favoured this result: the bee density, no competing food plants and a more preferred food plant species for the vectoring bees.

Our results showed an even distribution of the Prestop-Mix up to 100 m from the hives. Unfortunately, we were unable to measure larger distances, but the distribution of *Clonostachys rosea* by *B. impatiens* has been observed up to 150 m^34^. Wolf and Moritz^35^ showed that the mean foraging distance of *B. terrestris* was 267 m, whereas 40% of bumble bees foraged within a radius of 100 m. Although the bumble bees lost approximately 81% of the Prestop-Mix within the first 60 seconds of their flight^17^, the load on their hairs remained sufficiently high to guarantee distribution. The counting of bumble bees on the flowers and the bumble bee corbiculate pollen analysis revealed a low visitation rate. On average, we observed 1 bumble bee per 100 m when walking slowly along the strawberry rows. Strawberry pollen comprised approximately one-fifth of all the pollen gathered by bumble bees. This result is not surprising because bumble bees are food generalists, and *B. terrestris* is known to forage on several plant species simultaneously^23^,^36^,^37^. We believe that the primary inoculation made by bumble bees was enhanced by other flower visitors that served as secondary distributers of the biocontrol agent^13^. It seems that a high diversity of other pollinator species supports the success of the entomovector technology. The strawberry flowering occurs when there is almost no other wild bumble bees foraging because the young queens are still establishing their nests. However, the numbers of foraging honey bees, certain solitary bee species and different species of dipterans might be considerably high. In addition to the impact on the biocontrol activity, it is believed that the strawberry flowers benefit from the visits from pollinators that belong to different groups due to their behavioural displays on the flowers^38^. The added value from applying the entomovector technique derives from enhancing the pollination activity of the target crop. There are more than 200 stigmas in the strawberry flowers^39^,^40^, and each of them needs to be fertilised to achieve high quality fruits. Properly pollinated fruits not only are bigger but also have a longer shelf-life, which increase their commercial value^19^,^41^. In our experiment, we found no increase in fruit weight on insect-pollinated plots compared with those on the wind-pollinated plots. This result suggests that the cultivar ‘Sonata’ can cope with wind pollination alone.

The calculation of the total yield involves both the effects from the grey mould control and the increased insect pollination rate. Despite the lack of statistically significant differences between the total yield from the treated and not-treated plots, we observed an increase over the years when the bumble bee-mediated Prestop-Mix application was used. This stable positive trend could encourage farmers to use the entomovector technology in their strawberry cultivation, especially in organic production. The use of honey bees as vectors for biocontrol agents have already been accepted by Finnish growers^9^ and has been trialled by some Estonian strawberry growers. Using bumble bees as extra pollinators is going to be even more popular in Estonia. However, our results do not support a reliable yield increase, at least in the cultivar ‘Sonata’. The strawberry cultivars vary in their need for insect pollination. For instance, other frequently grown strawberries (cultivar Polka^20^, cultivar Elsanta^19^) significantly benefit from insect pollination.

The Prestop-Mix is an environmentally friendly plant protection product that is allowed in organic farms^42^. *Gliocladium catenulatum* has been considered safe for mammals, insects, and plants^43^. Because it is a natural soil fungus, wild organisms can come into contact with *G. catenulatum* almost everywhere through airborne soil dust. The treatment only increases the concentration on the flowers to achieve the pathogen-controlling effect. Although vectoring insects are exposed to extremely high doses when passing through the dispenser, The Prestop-Mix does not cause any negative effects on the longevity nor on the foraging activity of the pollinators^10^,^44^,^45^. Mommaerts *et al*.^44^. showed an increased mortality in vectoring bumble bees only in the case of a one-way dispenser because the bees moved in and out of the hive through the powder, and thus, larger amounts of the preparation were taken into the hive. In the case of a two-way dispenser, the bees move into the hive through an empty corridor, and thus, the hive remains unpolluted. In addition, at the individual level, forager bumble bees lose 81% of the original load within the first 60 seconds of flight^17^, and they continue to lose the powder over time. However, after a longer exposure with no chance to get the biopesticide off of the hairs, the bees’ cuticle became damaged, and the water loss rate increased. This undesired outcome was most likely due to the presence of kaolin in the composition of the biopesticide^45^. The described effect still would not affect the longevity of a commercial bumble bee colony, which is bought for fulfilling a short-term task.

In conclusion, our experiment demonstrates the reliability of the biocontrol agent application using bumble bees as vectors for the control of grey mould. *B. terrestris* bumble bees proved to be effective vectors for biocontrol agents in open field strawberry plantations throughout the three studied years in northern climatic conditions. Further research is required to extend the applicability of this technique in different spatiotemporal scales.

## Materials and Methods

### Inoculum, vectors and dispensers

A fresh commercial preparation of Prestop-Mix (*G. catenulatum* Strain J1446; Verdera Oy, Espoo, Finland) was used in all the assays. The carrier substance of the preparation was kaolin^45^, and the *G. catenulatum* concentration was >10^7^ colony forming units (CFUs)/g of the product^46^.

Bumble bee (*B. terrestris*) Multi Hives for outdoor use were obtained from Biobest (Biobest NV, Westerlo, Belgium). Multi Hive is a large, weather-resistant, polystyrene hive for outdoor use and consists of three separated compartments that each contains a colony with a queen and more than 350 workers. All the hives were equipped with two-way dispensers (with or without Prestop-Mix)^17^. In a two-way dispenser, the bumble bees pass through the dispenser tray that contained the biopesticide on their way out, but the inward movement occurred via an empty tunnel to avoid the contamination of the internal parts of the hives with the product^44^.

### Strawberry fields and plots

The study was conducted in two strawberry *F. x ananassa* cultivar ‘Sonata’ production fields in Southern Estonia during a three-year period (2012–2014). The perennial strawberry fields were established in August 2010 (Viljandi County, Polli, 2.0 ha) and May 2012 (Tartu County, Rõhu, 0.5 ha). In both fields, strawberries were planted in single rows, 6 plants per meter, and each row was 0.6 × 125 m. Both strawberry fields were surrounded by orchards with fruit trees and berry cultures. Open crop fields were present in the Rõhu experimental area within a 0.5-km foraging radius of the bumble bees. Both fields were established according to the suggestions needed for organic strawberry cultivation, and no plant protection chemicals (other than the experimental one) were used in either of the fields during the experimental years.

The strawberry cultivar used in the experiment was ‘Sonata’, which is one of the most commonly grown strawberry cultivars in Estonia. The flowering period started between May 25–28 and ended between June 10–14 during the three years of the experiments (2012, 2013 and 2014).

Eight experimental plots were marked on the rows at 5–25 m from the hives. Each plot consisted of 12 strawberry plants. Four plots were constantly covered with a neutral coloured anti-insect net (mesh size 0.44 × 0.77 mm, 9–10% shading, Retificio Padano, Ospitaletto, BS, Italy), which allowed wind pollination throughout the flowering period. The net allowed an adequate ventilation, but it isolated flowers from both pollinating insects and vectoring bumble bees. On the remaining plots, in addition to the wind pollination, the strawberry flowers were available for insect pollination and were also treated with the Prestop-Mix. Data loggers (HAXO-8 Temperature & Humidity Logger, MicroDAQ.com, NH 03229 USA) placed at canopy height on plots covered and non-covered with nets were used to monitor the relative humidity and air temperature. No significant differences in microclimate conditions were found between the covered and non-covered plots (RH: *t* = 0.05, *df* = 23, *P* = 0.95; Air temperature: *t* = −0.38, *df* = 23, *P* = 0.7).

### Experimental setup

**Figure 3:**
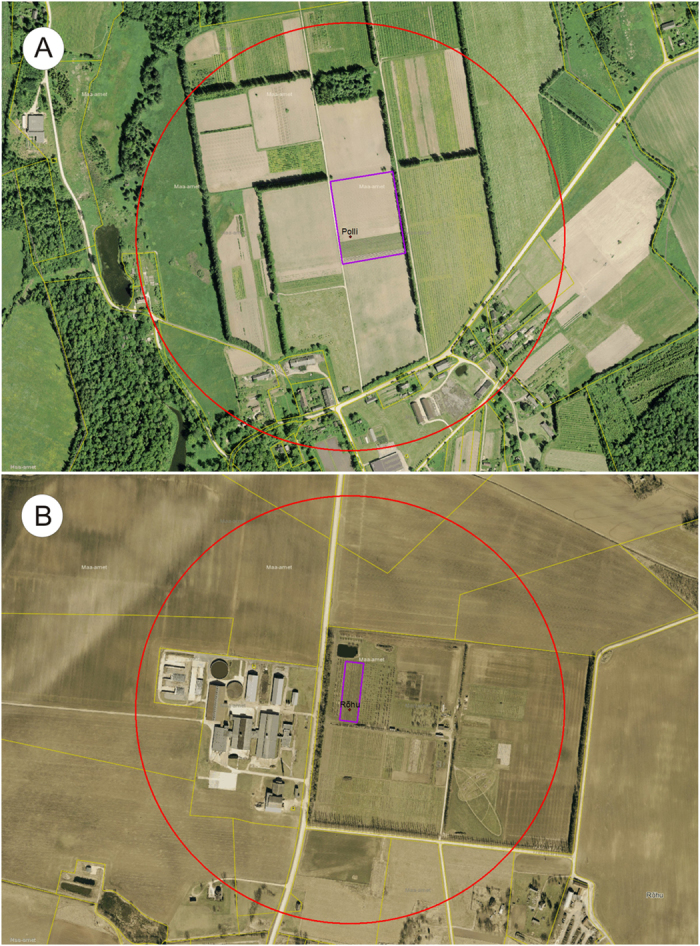
Maps of the strawberry fields (**A**) in Polli (Viljandi County, Estonia) and (**B**) Rõhu (Tartu County, Estonia). Strawberry fields are surrounded with purple rectangles; the dots in the fields represent the position of the hives on the field with the name of the experimental area, and the red circles delineates the 500-m area which represents the most likely foraging area of *B. terrestris* surrounding the hives. All the hives were equipped with two-way dispensers (BioPest), but the biocontrol Prestop-Mix preparation was used first in Polli (2012) and, subsequently, in Rõhu (2013, 2014). In 2013 and 2014 in Polli only pollination efficiency without the pathogen control was assessed. The backdrop maps are created using X-GIS(6) from Estonian Land Board mapserver (Copyright: Estonian Land Board 2016).

The same protocol was followed over the three seasons and in both fields. Two bumble bee triple hives (=6 colonies) were taken to the strawberry fields before the flowering period started. The colony density in Rõhu was 12 colonies per ha and in Polli 3 colonies per ha. The bumble bee nest entrances were kept closed until the flowering began, i.e., 1–2 flowers per plant were open, on average. The hives were positioned inside the fields, at 10 m from the closest field edge, as shown in the site maps (Fig. 3).

For measuring the effect of the biopesticide vectored by bumble bees, 6 g of Prestop-Mix was added to the dispenser before the first opening of the hive entrances (Polli 2012, Rõhu 2013 and 2014). The Prestop-Mix in the dispenser was examined daily for the amount and condition of the powder. At least every second day, all the remaining powder was removed, the dispenser was cleaned and refilled with fresh powder. The procedure was repeated when the powder was clumped due to a high air humidity. For measuring the effect of pollination only (Polli 2013 and 2014), the same bumble bee hives and dispensers with no biopesticide were used.

The fruit collecting period started on approximately June 26–28 and lasted until July 8–11 of the studied years. We collected strawberry fruits every third day, i.e., in the same way as commercial producers do. We separated and counted healthy and infected fruits, and the data were used to calculate the grey mould infection rate. The weight of ten randomly selected fruits was measured to determine the effect of pollination. The yield of healthy berries was summarised over all the collecting days per plot.

### Dispersal distance and measurement of *Gliocladium catenulatum* in flowers by quantitative real-time PCR

The distribution evenness of the biocontrol agent over the strawberry fields was estimated by collecting fresh strawberry flowers from distances of 0, 25, 50, 75 and 100 m from the hives (Rõhu field in 2013). At each distance, we had four replicate sampling plots. The plots were located along four transects that originated from the hives. The plots were open for insect visits. Ten flowers from each replicate plot were collected separately into minigrip plastic bags. The flowers were collected on 4 different days throughout the flowering period. All flowers were placed on the ice immediately after collection and kept at −20 °C until their analysis. The flower collection was made on days favourable for bumble bees foraging.

From each distance and each time point, we analysed 4 flowers using the qPCR method as developed by Dreo and Cokl^47^. In brief, the flowers were individually washed, and then the DNA was extracted with the PowerPlant® Pro DNA Isolation Kit as well as a combination of zirconia/silica beads (0.5 mm) and glass beads (0.1 mm) in the bead beating. The TaqMan-based qPCR was performed in triplicate. The reaction volume of 10 μl consisted of 5 μl of GoTaq® Probe qPCR Master Mix, 0.15 μl of TaqMan MGB probe, 0.6 μl of 10 μM forward primer (Invitrogen), 0.6 μl of 10 μM of reverse primer (Invitrogen), 1.65 μl of nuclease-free water and 2 μl of DNA. This mix was loaded into 96-well format Microseal PCR plates (Bio-Rad) and monitored on a CFX96™ Real-Time PCR detection system (Bio-Rad). In each amplification, we used a standard curve with several dilution series (101 to 106) of spores of *G. catenulatum*; a no template sample was used as negative control.

### Flower visitors

The numbers of flower-visiting insects were counted on days suitable for bee foraging using the transect walk method in the Rõhu field in 2013 throughout the strawberry flowering. The insect counts were made twice per day at randomly selected times on 100 m transects (N = 4) divided into 10 m sections at distances 0, 25, 50, 75 and 100 m from the hives.

### Bumble bee foraging on strawberry flowers

The corbiculate pollen pellets of homing bumble bees were gathered from each hive, each year and each location. The bumble bees were caught into a queen marking cage, immobilised by gently pushing her against a net, and the corbiculate pollen was removed using wooden tooth sticks; after this procedure, the bee was released. The pollen pellets were air dried and later acetolysed for plant species identification with light microscopy (Olympus CX 31 RBSF) using the 400x magnification. Strawbrry pollen grains have particulate features that make them easily identifiable from other plant species, even among genera of the family Rosacea, where it belongs. In total, 960 pollen samples were analysed.

### Climatic data

Rainfall and air temperature data were obtained from the Tartu-Tõravere meteorological station of the Estonian Weather Service (Tõravere Weather Station of the Estonian Weather Service).

### Statistical analysis

A Kruskal-Wallis test was used to compare the percentage of strawberry pollen gathered by bumble bees in the different fields and years. The analysis was performed with all the five field and year combinations; however, an extremely different year was excluded from the analysis. A non-parametric one way ANOVA and a bootstrapping test were used for comparing the distribution of the Prestop-Mix over the fields. The numbers of foraging bumble bees per 10-m transect section at the beginning and end of the flowering period and at five distances (0, 25, 50, 75, 100 m) from the hive were compared using a generalised linear model with the logarithm link function (Poisson model). In this model, we also considered the confounding effect of daytime and the random transect effect. The effect of insect pollination on the mean weight of ten fruits was tested using a generalised linear model, and the random effects of field by year combination and plots were also considered. The treatment effect on the grey mould infection rate was studied using a generalised linear model with the logit link function (logistic model), and the effects of year, year by treatment interaction and the random effect of plots were also taken into account. The treatment effect in the different years was tested with properly defined contrasts. The treatment and pollination effects on all the strawberry fields were tested separately using general linear models that considered the effect of year/field and treatment/pollination by year/field interaction effects. The treatment or pollination effect in different years or fields was tested with properly defined contrasts. The random effects in different models took into account the potential correlation between the measurements made in the same field/plot/transect/year and, thus, these measurements were not considered as independent replicates (the analysis considered them as potential pseudoreplicates).

The values in the text and figures are presented as the mean ± standard errors, and the results were considered statistically significant at p ≤ 0.05. All statistical the analyses were performed with the SAS 9.4 statistical package (SAS Institute Inc., Cary, NC, USA), except for those used in the Prestop-Mix distribution analyses, which were made in SPSS 22.0.

## Additional Information

**How to cite this article**: Karise, R. *et al*. Reliability of the entomovector technology using Prestop-Mix and *Bombus terrestris* L. as a fungal disease biocontrol method in open field. *Sci. Rep.* **6**, 31650; doi: 10.1038/srep31650 (2016).

## Figures and Tables

**Table 1:** Weather data of the strawberry flowering and berry picking period (2012–2014) with the long-term monthly average.

	Temperature (°C)			Rainfall (mm)			Number of rainy days		
	May	June	July	May	June	July	May	June	July
2012	12	13	18	76	89	69	7	10	9
2013	14	18	17	73	35	59	9	9	9
2014	**12**	**14**	20	**90**	**134**	78	**16**	**18**	8
Long-term average	11	15	16	53	69	76			

The long-term average is based on data obtained from the period 1981–2010.
